# Acute Augmented Effect of Virtual Reality (VR)–Integrated Relaxation and Mindfulness Exercising on Anxiety and Insomnia Symptoms: A Retrospective Analysis of 103 Anxiety Disorder Patients With Prominent Insomnia

**DOI:** 10.1002/brb3.70060

**Published:** 2024-09-30

**Authors:** Hao Zhou, Cuijie Chen, Jinxi Liu, Changhe Fan

**Affiliations:** ^1^ Department of Psychiatry The Affiliated Guangdong Second Provincial General Hospital of Jinan University Guangzhou P. R. China

**Keywords:** anxiety disorders, insomnia, mindfulness, relaxation, retrospective analysis, virtual reality

## Abstract

**Background:**

Anxiety disorder is one of the most common mental disorders and often accompanied with sleep disturbance which can in turn exacerbate anxiety symptoms, creating a vicious cycle. In addition to psychopharmacological therapy, the effectiveness of psychotherapy as cognitive behavioral therapy (CBT) for treating anxiety disorders and insomnia has been well documented and widely accepted, but it is labor‐intensive and costly. However, virtual reality (VR)–integrated CBT may improve this condition but needs more evidences to support its extensive application in routine clinical practice.

**Objectives:**

This explorative study was aimed to conduct a retrospective analysis to evaluate the acute (2 weeks) augmented effect of VR‐integrated relaxation and mindfulness exercising in improving anxiety and insomnia symptoms for patients who were diagnosed with anxiety disorders and concurrently with prominent insomnia symptoms and admitted to the Department of Psychiatry, The Affiliated Guangdong Second Provincial General Hospital of Jinan University during January 2021 to June 2021.

**Methods:**

All patients who were admitted to the department of psychiatry during January 1, 2021 to June 30, 2021 were screened with inclusion criteria and exclusion criteria, and the sociodemographic and clinical data of those included patients were collected from the electronic medical record system of the hospital using a self‐designed case report form (CRF). Subjects who were administrated with medication alone were designated as conventional group, and those receiving treatment of medication combined with VR‐integrated CBT (VR relaxation and mindfulness exercising) as VR group. The baseline and 2‐week posttreatment data were compared between the two groups.

**Results:**

In total, there were 103 patients (70 female, 68%) included in the study. Among all, 68 (66.02%) were designated as the “VR group,” and 35 (33.98%) as the “conventional group.” The majority of patients (67%) were diagnosed with generalized anxiety disorder (GAD). Twenty‐three (22.3%) patients had a comorbid diagnosis with primary insomnia, and insomnia was just one of the accompanying symptoms with anxiety for the rest 80 subjects. No statistically significant differences were found between VR and conventional groups in all baseline sociodemographic and clinical characteristics except for occupation. There were statistically significant differences for the remission rates of anxiety symptoms or insomnia symptoms and reduction of Hamilton Anxiety Rating Scale or Insomnia Severity Index total scores between conventional and VR groups. Greater remission rates or score reductions were found in VR group than in conventional group either for anxiety or for insomnia. Robust differences still existed when controlled for the variable “occupation.”

**Conclusions:**

Two‐week augmented VR‐integrated relaxation and mindfulness exercising is acutely beneficial for relieving both anxiety and insomnia symptoms and worth being recommended for routine clinical practice. Further prospective and randomized study compared to traditional CBT to explore its acute and long‐term effect on anxiety and insomnia is needed.

## Graphical abstract

This explorative and retrospective study evaluated the effect of VR‐integrated relaxation and mindfulness exercising in improving anxiety and insomnia symptoms in patients with anxiety disorders and prominent insomnia and indicated that 2‐week augmented VR‐integrated relaxation and mindfulness exercising is acutely beneficial for relieving both anxiety and insomnia.

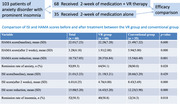

## 1 Introduction

Mental illness is one of the top causes of global health‐related burden, with anxiety disorder ranked as the 24th most common disorder in the Global Burden of Disease (GBD) in 2019 (GBD 2019 Diseases and Injuries Collaborators 2020). A recent epidemiological survey conducted in China revealed that anxiety disorder is one of the most common mental disorders with 7.6% of lifetime prevalence among general population (Huang et al. 2019) and the COVID‐19 pandemic adds many risk factors for the development of mental illness, with a 25.6% increase in global cases of anxiety disorders during 2020 (COVID‐19 Mental Disorders Collaborators 2021). Patients with anxiety disorders are often accompanied with sleep disturbance, such as insomnia symptom or comorbid with insomnia disorder, and these sleep‐related symptoms can in turn exacerbate anxiety symptoms, creating a vicious cycle (Papadimitriou and Linkowski 2005; Staner 2003). In addition to psychopharmacological therapy, the effectiveness of psychotherapy as cognitive behavioral therapy (CBT) for treating anxiety disorders (Trauer et al. 2015) and insomnia (Zhang et al. 2019) has been well documented and widely accepted, but it is labor‐intensive and costly, and the capability of practitioners varies, which makes it difficult to ensure treatment efficacy and consistency. However, with the development of technology, many new technologies have been brought into the clinical practice, and virtual reality (VR), as one of the cutting‐edge technologies, has a promising future in the treatment of psychiatric disorders (Freeman et al. 2017; Jerath and Beveridge 2021). VR is defined as a human–machine interface that allows users to project themselves into a computer‐generated virtual environment, where specific objectives can be achieved (Zhang 2014). Integrating VR technology with CBT therapy, including relaxation therapy and mindfulness therapy, can provide a more standardized and labor‐saving process for CBT and may greatly improve the efficacy of treatment for anxiety disorder and insomnia. However, this integrated process has not been widely applied in clinical practice currently and still needs further evidence of its efficacy. This explorative study was aimed to conduct a retrospective analysis to evaluate the acute (2 weeks) effect of VR‐integrated CBT in improving anxiety and insomnia symptoms for patients who were diagnosed with anxiety disorders and concurrently with prominent insomnia symptoms and admitted to the Department of Psychiatry, The Affiliated Guangdong Second Provincial General Hospital of Jinan University from January 2021 to June 2021.

## 2 Methods

### 2.1 Subjects and Data Collection

All patients who met all items of the following inclusion criteria and did not meet any item of the exclusion criteria were included in this study, and their sociodemographic and clinical data from the electronic medical record system of the hospital were collected:

#### 2.1.1 Inclusion Criteria

(1) Diagnosed with any subtype of anxiety disorders according to the diagnostic criteria of Diagnostic and Statistical Manual of Mental Disorders, 4th Edition, Text Revision (DSM‐IV‐TR). (2) Age ≥14 years and admitted to the Department of Psychiatry, The Affiliated Guangdong Second Provincial General Hospital of Jinan University during January 1, 2021 to June 30, 2021, and stayed on treatment (medication alone or medication combined with VR‐integrated relaxation and mindfulness exercising) for at least 2 weeks continuously either at inpatient ward or outpatient department after discharge. (3) Had been rated both with Hamilton Anxiety Rating Scale (HAMA) and Insomnia Severity Index (ISI) at the day of admission (baseline) and the day after 2 weeks of treatment (posttreatment). (4) Had current complaints of prominent insomnia symptoms or comorbid diagnosis of primary insomnia according to DSM‐IV‐TR at admission. And the baseline total score of ISI ≥ 14 or the baseline Item 4 (insomnia) score of HAMA ≥ 3. (5) Administrated with no other systematic psychotherapy or neuromodulation therapy such as repetitive transcranial magnetic stimulation (rTMS) and transcranial direct current stimulation (tDCS) except for psychotropic medication alone or psychotropic medication combined with VR‐integrated relaxation and mindfulness exercising.

#### 2.1.2 Exclusion Criteria

(1) Diagnosed with severe and unstable physical illness and needed intervention from other clinical department. (2) Diagnosed with drug or alcohol‐related disorder. (3) Incomplete data especially without posttreatment data.

#### 2.1.3 Data Collection

All patients who were admitted to the department of psychiatry from January 1, 2021 to June 30, 2021 were screened with inclusion criteria and exclusion criteria, and then a self‐designed case report form (CRF) was used to collect sociodemographic and clinical data of all included subjects from the electronic medical record system of the hospital. Patients who were administrated with medication alone were designated as Conventional Group, and those receiving treatment of medication combined with VR‐integrated relaxation and mindfulness exercising as VR group.

### 2.2 Statistical Analysis

Data analysis was performed using SPSS 26.0 statistical software. Values of quantitative variables were denoted as mean (standard deviation, SD) and were compared by independent sample *t*‐test or by Mann–Whitney *U*‐test. Categorical variables were expressed as *n* (%) and compared by the Pearson chi‐square test or Fisher's exact test.

### 2.3 Ethical Statement

All subjects had previously signed informed consent form at the day of admission agreeing to the future utilization of their medical data anonymously for medical research. Moreover, the study protocol was reviewed and approved by the Ethics Committee of Guangdong Second Provincial General Hospital (No.: 2021‐KZ‐096‐02). All procedures during data collection and analysis were conducted in accordance with the Declaration of Helsinki (as revised in 2013).

## 3 Results

### 3.1 Baseline Sociodemographic and Clinical Characteristics

Altogether, there were 103 patients (70 female, 68%) included in the study. Among all, 68 (66.02%) patients received both psychotropic medication and VR‐integrated relaxation and mindfulness exercising treatment and were designated as the “VR group,” and 35 (33.98%) patients received medication alone and were designated as the “conventional group.” The majority of patients (67%) were diagnosed with generalized anxiety disorder (GAD). As for the relationship between anxiety and insomnia, 23 (22.3%) patients had a comorbid diagnosis with primary insomnia, and insomnia was just one of the accompanying symptoms with anxiety for the rest 80 subjects. Moreover, 33 (32%) patients had another psychiatric diagnosis, including another subtype of anxiety disorder except for primary insomnia. Although comorbid severe psychiatric disorder was not an exclusion criterion, there were only two subjects comorbid with bipolar disorder and five with major depressive disorder. The baseline sociodemographic and clinical characteristics of all subjects and comparison between VR and conventional groups are shown in Table 1. No statistically significant differences were found between VR and conventional groups in all baseline sociodemographic and clinical characteristics except for “occupation.”

**TABLE 1:** Baseline characteristics of all subjects and comparison between subjects in virtual reality (VR) and conventional groups.

Variable	Total (*n* = 103)	VR group (*n* = 68)	Conventional group (*n* = 35)	*t*/*χ* ^2^	*p* value
Gender, *n* (%)					
Female	70 (68.0)	49 (72.1)	21 (60.0)	1.543	0.214
Male	33 (32.0)	19 (27.9)	14 (40.0)		
Age (years), M (SD)	44.83 (15.47)	43.56 (15.08)	47.31 (16.13)	1.169	0.245
Weight (kg), M (SD)	59.09 (10.23)	58.92 (10.43)	59.43 (9.97) 56.005	0.239	0.812
Educational level, *n* (%)					
≤Junior high school	51 (49.5)	34 (50.0)	17 (48.6)	0.019	0.891
≥Senior high school	52 (50.5)	34 (50.0)	18 (51.4)		
Years of education, M (SD)	10.65 (4.31)	10.50 (4.39)	10.94 (4.19)	0.492	0.624
Occupation, *n* (%)					
Unemployed or student	54 (52.4)	30 (44.1)	24 (68.6)	5.540	0.019
Employed or retired	49 (47.6)	38 (55.9)	11 (31.4)		
Duration of anxiety disorder (months), M (SD)	48.99 (76.77)	45.03 (80.82)	56.66 (68.66)	0.726	0.469
Recurrent anxiety disorder, *n* (%)	58 (56.3)	38 (55.9)	20 (57.1)	0.015	0.903
Days of hospitalization, M (SD)	12.76 (5.87)	12.57 (4.86)	13.11 (7.52)	0.441	0.660
Readmission, *n* (%)	15 (14.6)	7 (10.3)	8 (22.9)	2.931	0.087
Subtype of anxiety disorder, *n* (%)					
GAD	69 (67)	45 (66.2)	24 (68.6)	0.076	0.963
PD	18 (17.5)	12 (17.6)	6 (17.1)		
Other subtypes^a^	16 (15.5)	11 (16.2)	5 (14.3)		
Anxiety‐insomnia relationship, *n* (%)					
Comorbid with primary insomnia	23 (22.3)	17 (25.0)	6 (17.1)	0.822	0.364
Accompanied with insomnia symptom	80 (77.7)	51 (75.0)	29 (82.9)		
Comorbid psychiatric diagnosis except insomnia disorder, *n* (%)	33 (32.0)	22 (32.4)	11 (31.4)	0.009	0.924
Number of comorbid physical diseases, M (SD)	2.17 (2.26)	2.07 (2.19)	2.37 (2.39)	0.633	0.528
Number of categories of psychotropic drugs used, M (SD)	2.47 (0.54)	2.47 (0.56)	2.45 (0.51)	−1.19	0.905
Total number of psychotropic drugs used, M (SD)	3.78 (1.24)	3.81 (1.19)	3.71 (1.34)	−0.366	0.715
Baseline HAMA score, M (SD)	22.01 (7.23)	22.28 (7.28)	21.49 (7.22)	−0.526	0.600
Baseline ISI score, M (SD)	21.01 (3.23)	21.19 (3.21)	20.66 (3.29)	−0.794	0.429
Dose of AD equivalent to duloxetine (mg)^b^, M (SD), *n* = 96	51.17 (27.76)	51.47 (26.53)	50.54 (30.64)	−0.153	0.879
Dose of BZD and HPN equivalent to diazepam (mg)^b^, M (SD), *n* = 99	15.29 (23.21)	13.71 (22.93)	18.18 (23.77)	0.914	0.363
Dose of BZD equivalent to diazepam (mg)^b^, M (SD), *n* = 96	12.64 (23.66)	10.55 (23.16)	16.83 (24.44)	1.230	0.222
Dose of HPN equivalent to diazepam (mg)^b^, M (SD), *n* = 58	5.17 (1.23)	5.06 (0.89)	5.42 (1.77)	0.805	0.430
Dose of AP equivalent to olanzapine (mg)^b^, M (SD), *n* = 59	3.75 (2.51)	3.57 (2.41)	3.59 (2.76)	0.028	0.977

Abbreviations: AD, antidepressant; AP, antipsychotics; BZD, benzodiazepines; GAD, generalized anxiety disorder; HAMA, Hamilton Anxiety Rating Scale; HPN, hypnotics; ISI, Insomnia Severity Index; M (SD), mean (standard deviation); PD, panic disorder.

^a^Included six patients with obsessive compulsive disorder (OCD), two with posttraumatic stress disorder (PTSD) and eight with acute anxiety state.

^b^Dose equivalence was calculated according to conversion tables in literatures (Hayasaka et al. 2015; Inada and Inagaki 2015).

### 3.2 Treatment Procedure

All patients were treated as clinically needed with psychotropic drug monotherapy, including antidepressants, benzodiazepines, non‐BZD hypnotics, antipsychotics, or combinative therapy (59 subjects received low doses of antipsychotics to improve anxiety or insomnia symptoms). Subjects in the conventional group received medication only for at least 14 consecutive days, and those in the VR group additionally received VR‐integrated relaxation and mindfulness exercising therapy for one session (about 15 min for relaxation and 15 min for mindfulness), twice a day (once in the morning and another in the afternoon) for at least 14 consecutive days.

VR‐integrated relaxation and mindfulness exercising therapy were provided by Med‐Vision Virtual Reality Mental Health Training System (Hangzhou Xinjing Technology Co. Ltd) which adopts VR and EEG biofeedback technology and combines relaxation therapy, music therapy, mindfulness meditation, hypnotherapy, and so forth to build an immersive VR environment for programmed training and monitor the physiological and psychological indicators of the subjects in real time. VR relaxation training and VR mindfulness exercising modalities contained a total of 14 different virtual scenarios, including grassland, bamboo forest, garden, lotus pond, open country, stream, high mountains, and north Xinjiang scenery.

All subjects in VR group were arranged to conduct relaxation and mindfulness exercising in a specialized psychotherapy room that can accommodate therapy for 10–12 patients at the same time. A physical therapist assisted each subject to sit in a chair, adjusted the equipment, selected VR sceneries and training modules (relaxation or mindfulness), helped the subject put on VR headgear, briefly guided them on the main content of VR training, and told them to ask for help at any time if they have problems during exercising. And then the subject was immersed in VR sceneries and engaged in relaxation or mindfulness exercising following the verbal guidance from VR headgear.

All patients were assessed using clinical rating scales such as HAMA, ISI, and the Eysenck Personality Questionnaire (EPQ) and interviewed by a psychiatrist at the day of admission. Treatment principles and specific methods, including pharmacotherapy and non‐pharmacotherapy, including VR‐integrated relaxation and mindfulness exercising therapy, were explained and recommended to all patients by psychiatrists after clinical assessment. Individualized medication regimens and whether to take VR‐integrated relaxation and mindfulness exercising therapy or not were made by psychiatrists and patients together on the basis of the clinical assessment and patients’ preference. The number of medication categories and total number of psychotropic drugs are shown in Table 1. Equivalent doses of antidepressants (to duloxetine), benzodiazepines (to diazepam), hypnotics (to diazepam), and antipsychotics (to olanzapine) were calculated according to conversion tables in the literatures (Hayasaka et al. 2015; Inada and Inagaki 2015). There were no statistically significant differences for average doses of above psychotropic drugs between conventional and VR groups.

### 3.3 Outcome Measure

The baseline and posttreatment total scores of HAMA and ISI scales were collected. Remission of anxiety symptoms or insomnia symptoms was defined as total HAMA score ≤6 or total ISI ≤7. The remission rates and reduction of HAMA or ISI total scores 2 weeks from baseline were calculated and compared between conventional and VR groups to explore the effects of VR‐integrated CBT in improving anxiety and insomnia symptoms. As shown in Table 2, the acute (2 weeks) remission rates of anxiety symptoms or insomnia symptoms for the whole group were 89.3% and 50.5%, respectively. There were statistically significant differences for the remission rates of anxiety symptoms or insomnia symptoms and reduction of HAMA or ISI total scores between conventional and VR groups. Greater remission rates or score reductions were found in VR group than in conventional group either for anxiety or for insomnia. Robust differences still existed when controlled for the variable “occupation.”

**TABLE 2:** Comparison of Insomnia Severity Index (ISI) and Hamilton Anxiety Rating Scale (HAMA) scores before and after treatment between the virtual reality (VR) and conventional groups.

Variable	Total (*n* = 68)	VR group (*n* = 68)	Conventional group (*n* = 35)	*t/χ* ^2^	*p* value
HAMA score (baseline), mean (SD)	22.01 (7.23)	22.28 (7.28)	21.49 (7.22)	−0.562	0.600
HAMA score (after 2 weeks), mean (SD)	3.28 (4.18)	1.91 (2.88)	5.94 (5.00)	5.195	0.000
HAMA score reduction, mean (SD)	18.73 (7.05)	20.37 (6.84)	15.54 (6.40)	−3.465	0.001
Remission rate of anxiety, *n* (%)	92 (89.3)	64 (94.1)	28 (80.0)	4.828	0.028
ISI score (baseline), mean (SD)	21.01 (3.23)	21.19 (3.21)	20.66 (3.29)	−0.794	0.429
ISI score (after 2 weeks), mean (SD)	6.01 (4.25)	4.76 (4.00)	8.43 (3.69)	4.518	0.000
ISI score reduction, mean (SD)	15.00 (5.20)	16.43 (5.20)	12.23 (3.98)	−4.186	0.000
Remission rate of insomnia, *n* (%)	52 (50.5)	40 (58.8)	12 (34.3)	5.566	0.018

Abbreviation: SD, standard deviation.

## 4 Discussion

Anxiety disorders which are characterized by excessive fear and anxiety and related behavioral disturbances are among the most frequently encountered psychiatric disorders (Diemer et al. 2014.). Insomnia is one of the most commonly concomitant symptoms in anxiety disorder in addition to its core symptoms. Insomnia and anxiety have common pathophysiological mechanisms and are prone to comorbidity (Glidewell, McPherson Botts, and Orr 2015). CBT and/or medication is recommended treatments for both anxiety disorder and insomnia disorder. In recent years, digital techniques, such as VR, have been employed in tandem with more traditional psychological interventions (Ma et al. 2023) and especially emerged as an attractive alternative for the treatment of anxiety and related disorders (Fernández‐Álvarez, Di Lernia, and Riva 2020). Previous studies have shown positive effects of VR‐based psychological intervention on anxiety or insomnia separately while few studies focusing on them simultaneously.

The current explorative study aimed to evaluate the acute augmented effects of VR‐based CBT (relaxation training and mindfulness exercising) on anxiety and insomnia symptoms in patients of anxiety disorder with prominent insomnia through retrospective analysis of real‐world clinical data. The results showed that the baseline sociodemographic and clinical characteristics, including gender, age, education, weight, diagnosis subtype, relationship with insomnia, duration of disease, recurrence, readmission, days of hospitalization, HAMA and ISI scores, number, category, and dosage of psychotropic drugs, were comparable between subjects in VR group (VR intervention + medication) and conventional group (medication alone) except for “occupation.” After 2 weeks of consecutive treatment, there were statistically significant differences for the remission rates of anxiety symptoms or insomnia symptoms and reduction of HAMA or ISI total scores between conventional and VR groups. Greater remission rates or score reductions were found in VR group than in conventional group either for anxiety (94.1% vs. 80.0%) or for insomnia (58.8% vs. 34.3%). Robust differences still existed when controlled for the variable “occupation.” These results indicate that VR‐integrated relaxation and mindfulness exercising has acute augmented effect in improving both anxiety and insomnia symptoms.

The result of the present study is similar to previous studies. de Zambotti et al. (2022) found that VR‐guided meditation and paced breathing resulted in acute autonomic and cortical modulation and therefore improved insomnia. Pan et al. (2022) used VR therapy (VRT) to treat psychological problems in four cases of first‐line healthcare professionals during the COVID‐19 outbreak for a week and found scores of GAD‐7 and the Athens Insomnia Scale reduced 52.17% and 67.44%, respectively. A single, short VR‐GR (guided relaxation) session showed transient reductions in pain intensity, pain unpleasantness, and anxiety in children and adolescents with acute postoperative pain (Olbrecht et al. 2021). A recent prospective, experimental, nonrandomized controlled trial showed evidence of immersive VR's capability to reduce state anxiety in OT (occupational therapy) students preparing for clinical practical exams (Concannon, Esmail, and Roduta Roberts 2020). Other studies demonstrated that VRT and counseling reduced students public speech anxiety (Sarpourian et al. 2022), and VR meditation significantly reduced subjective reports of anxiety and increased Alpha power in anxious participants (Tarrant, Viczko, and Cope 2018).

Although the specific psychological intervention methods used are not exactly the same, the results in the current study and previous studies indicate that VR‐based psychological intervention is beneficial in improving anxiety and insomnia symptoms. Effect of psychological intervention (e.g., CBT) in treating anxiety and insomnia disorder has been well documented. VR is a computer‐generated simulation that recreates or imitates a realistic environment via sensations delivered to a person via a headset and/or other devices (Park et al. 2019). As an innovative method, VR‐integrated psychological intervention might represent an increasingly important addition to standard CBT (Diemer et al. 2014). The advantages of VR‐CBT include increasing patients’ motivation and engagement, the use of safe environments, the interactivity possibilities, and the high level of personalization offered by VR (Bell et al. 2020). Insomnia is a condition of psychophysiological hyperactivation (referred to as hyperarousal), involving the upregulation of several biopsychological domains, including cognitive, emotional, autonomic (ANS), and central (CNS) nervous systems, both during the day and night (de Zambotti et al. 2022). Anxiety and insomnia have similar underlying pathophysiology such as ANS or CNS hyperactivation and conditioned responses, and progressive muscle relaxation training and mindfulness exercising may help patients modulate the condition and thus improve the symptoms. In this study, we compared the acute (2 weeks) effects in patients who received medication combining with VR‐integrated relaxation training and mindfulness exercising with those who received medication alone, and the result was in support with previous findings. In sum, this study has added new evidence supporting VR‐integrated relaxation, and mindfulness exercising is a beneficial and promising augmented method for treating anxiety and insomnia. As VR systems are now commercially available at relatively low cost as stand‐alone HMD, leading toward a wide use of VR in our daily lives (de Zambotti et al. 2022), VR‐integrated relaxation and mindfulness exercising is worth being recommended for routine clinical practice especially in mental healthcare areas.

Although positive findings were revealed for augmented effects of VR‐integrated relaxation and mindfulness exercising in treating anxiety and insomnia, there are several limitations in this study. First, this study is an explorative, retrospective case–control study in which data quality cannot be guaranteed as in a prospective study. Second, no control group receiving traditional CBT had been included; thus, placebo effects cannot be excluded. Third, patients who did not complete 14 days of consecutive treatment were not included in the study, so the results cannot reflect the effect of VR‐relaxation and mindfulness exercising shorter than 2 weeks. Fourth, in addition to data relevant to efficacy, no other relevant data, including safety, acceptability, psychophysiological assessment, and mindfulness level rating, were collected due to retrospective study. So the whole profile and possible mechanism of augmented VR‐relaxation and mindfulness exercising in relieving anxiety and insomnia cannot be fully illustrated. Finally, because the assignment to VR group was not randomized due to the retrospective nature of this study, there may be selection bias for VR group. Although the baseline sociodemographic and clinical characteristics were comparable between subjects in VR and conventional groups (except for occupation), other characteristics that were not assessed, including psychological traits and intellectual capabilities, may differ. Subjects who chose to take VRT were probably less nervous, more resilient, with higher intellectual capability, and more open to try new intervention (e.g., VRT) than those who chose not. Therefore, it cannot be ruled out that the higher efficacy in VR group was partly due to differences in psychological traits and intellectual capabilities of subjects between the two groups. For future research, more strictly designed, prospective, and randomized study compared to traditional CBT to explore the acute and long‐term effects of VR‐CBT on anxiety and insomnia is needed.

In conclusion, 2‐week augmented VR‐integrated relaxation and mindfulness exercising is acutely beneficial for relieving both anxiety and insomnia symptoms and worth being recommended for routine clinical practice. Further prospective and randomized study compared to traditional CBT to explore its acute and long‐term effect on anxiety and insomnia is needed.

## Author Contributions

**Hao Zhou**: conceptualization, investigation, methodology, data curation, validation. **Cuijie Chen**: conceptualization, investigation, methodology, validation, data curation. **Jinxi Liu**: writing—original draft, writing—review and editing, formal analysis, data curation, methodology. **Changhe Fan**: conceptualization, investigation, funding acquisition, writing—original draft, methodology, validation, writing—review and editing, formal analysis, project administration, supervision, data curation.

## Conflicts of Interest

The authors declare no conflicts of interest.

### Peer Review

The peer review history for this article is available at https://publons.com/publon/10.1002/brb3.70060.

## Data Availability

The data that support the findings of this study are available from the corresponding author upon reasonable request.
